# Sleep characteristics and inflammatory markers in women with post-traumatic stress disorder

**DOI:** 10.1186/s12888-023-04765-1

**Published:** 2023-04-20

**Authors:** Mary Sau Ling Yeh, Dalva Poyares, Ana Teresa D. D’Elia, Bruno M. Coimbra, Andrea Feijo Mello, Sergio Tufik, Marcelo Feijo Mello

**Affiliations:** 1 Department of Psychiatry, Universidade Federal de Sao Paulo (UNIFESP), Rua Major Maragliano, 241, São Paulo, 04017-030 SP Brazil; 2 Department of Psychobiology, Universidade Federal de Sao Paulo, Sao Paulo, Brazil

**Keywords:** Post-traumatic stress disorder, Polysomnography, Sleep, Cytokines, Inflammatory markers

## Abstract

**Introduction:**

Sexual violence is one of the most severe traumatic events. It is associated with a higher risk for post-traumatic stress disorder (PTSD) development. Sleep disturbances such as insomnia are frequently reported by PTSD patients and play a key role in the development and course of the disorder. Sleep disturbances are associated with higher levels of pro-inflammatory cytokines emphasizing the importance of sleep studies in individuals with PTSD.

**Objectives:**

To investigate the association between subjective and objective sleep measurements and PTSD symptoms with inflammatory markers in women with PTSD following sexual assault.

**Methods:**

In this longitudinal study fifty-seven women with PTSD were evaluated for sleep measurements and inflammatory markers. Participants completed the Clinician-Administered PTSD Scale, the Beck Depression Inventory, the Pittsburgh Sleep Quality Index (PSQI), and the Insomnia Severity Index. In addition, patients underwent full in-lab polysomnography and serum levels of interleukin (IL)-1β, IL-6, tumor necrosis factor (TNF)-α, and C-reactive protein (CRP) measurement. All assessments were performed at baseline and after one year. Patients received pharmacological and/or psychological interventions between baseline and one-year follow-up.

**Results:**

Despite improving PTSD symptoms severity and sleep quality (expressed in PSQI), we found an increase in the inflammatory markers IL-1β, TNF-α, IL-6 and CRP after one year of follow-up. These findings suggest that neurobiological processes may advance independently of PTSD symptoms. We found a significant increase in the levels of IL-1β and TNF-α associated with decreased slow-wave sleep (p = 0.019 and p = 0.018 respectively), IL-6 associated with arousal index (p = 0.024), and CRP associated with insomnia severity (p = 0.012), and sleep duration longer than 6 h per night (p < 0.001).

**Conclusions:**

Sleep impairments in PTSD may be associated with a gradual and persistent alteration in the immune system, resulting in a progressive inflammatory process. Our results suggest that sleep mechanisms are involved in this incident inflammatory process in young women with PTSD.

## Background

Nightmares and insomnia are common symptoms and are considered the hallmark of PTSD [1]; also, a growing body of evidence suggests that sleep disturbances are a component of the pathophysiology of PTSD. Sleep disturbances immediately after trauma are a risk factor for PTSD development [2, 3], and improved sleep quality has been associated with a reduction in the severity of PTSD symptomatology [4, 5]. Moreover, sleep disruption preceding traumatic exposure also predicts PTSD development [6, 7].

Sleep is a crucial physiological process controlled by several regulatory systems, including a complex system of neuroendocrine, neurotransmitters, and neuropeptides that can be influenced by inflammatory biomarkers in a bidirectional manner [8]. Evidence suggests an association between disturbed sleep and inflammatory cytokines [8–11]. A study demonstrated that acute total and short-term partial sleep deprivation elevated C-reactive protein (CRP) concentrations in healthy adult subjects [10]. A meta-analysis evaluating sleep disturbance, sleep duration, and inflammation in population-based samples found an association between sleep disturbance and long sleep duration (> 8 h/night) with increases in inflammatory biomarkers [11]. Moreover, studies suggest that the association between sleep disturbances and pro-inflammatory cytokines is more robust in women than in men [5, 13]. Suarez (2008) found a correlation between poor sleep quality and greater frequency of disturbed sleep symptoms with elevated levels of IL-6 and CRP in women, while such an association was not observed in men, demonstrating sex differences in the association between sleep and cytokines [14–16].

Research suggests an association between disturbed sleep and inflammatory cytokines [8–11] however, few studies have investigated inflammatory biomarkers in PTSD-related sleep disturbances. Heinzelmann et al. (2014) showed that military personnel with insomnia who reported sleep restoration after deployment had reduced CRP concentrations, decreased depression severity, and tended toward fewer PTSD symptoms [17]. Nevertheless, another study did not find a significant correlation between insomnia severity and inflammatory markers in women with PTSD [18]. Understanding the magnitude of this association, in which potential inflammation might be involved, has critical clinical applications for reducing PTSD symptom severity and physical comorbidities. Elevated levels of systemic pro-inflammatory cytokines are a risk factor for the development of cardiovascular disease, metabolic syndrome, and dementia observed in chronic PTSD and are better described among male veterans [19–21].

Therefore, we aimed to study the relationship between sleep quality and inflammatory markers in women with PTSD. We investigated sexually assaulted women with PTSD by measuring changes in sleep measurements and inflammatory biomarkers over one year.

## Methods

Fifty-seven women with PTSD were evaluated between October 2015 and October 2018. Inclusion criteria were age between 18 and 45 years, a history of sexual assault six months before study enrollment, and a diagnosis of PTSD according to DSM-5 criteria. Exclusion criteria were menopausal symptomatology, pregnancy, corticosteroid use, history of human immunodeficiency virus infection, acute or unstable clinical conditions, neurological disorders, and a lifetime history of bipolar, psychotic, or substance dependence or abuse (not in remission in the previous six months). Participants undergoing any psychological or psychiatric treatment were also excluded. The ethics committee of the Universidade Federal de Sao Paulo (UNIFESP) approved all clinical procedures (reference number 3.115.325). All participants provided written informed consent prior to the assessments.

The current study is part of a more extensive study protocol entitled “Post-traumatic stress disorder and neuroprogression in women following sexual assault: protocol for a randomized clinical trial evaluating allostatic load and aging process acceleration.” Detailed methods have been described previously [22]. The clinical trial of this study was registered at the Brazilian Clinical Trials Registry (registration number: RBR-3z474z, registration date: 28/09/2017).

**Fig. 1:**
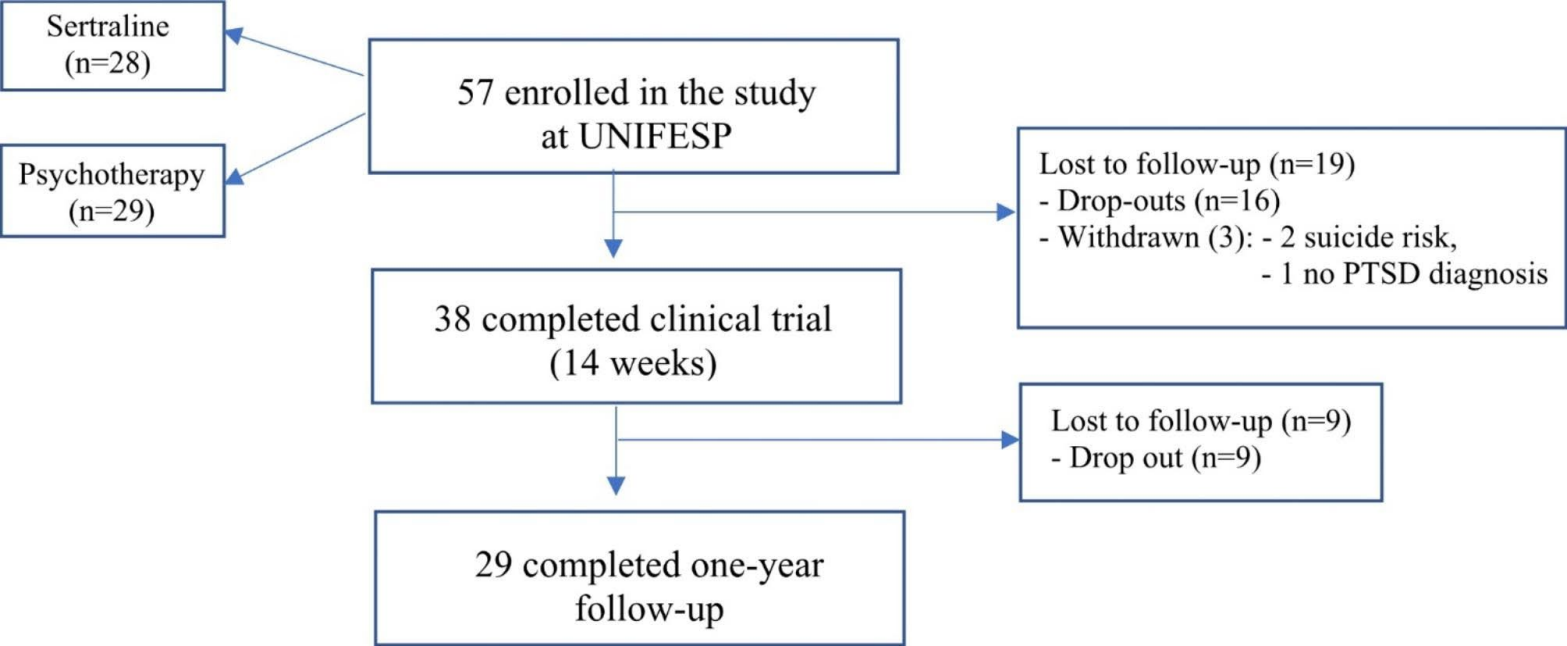
Study flowchart. UNIFESP: Universidade Federal de Sao Paulo; PTSD: Post-traumatic stress disorder

Trained psychiatrists and psychologists clinically assessed participants and underwent polysomnography (PSG) and blood collection at baseline and one-year follow-up. Participants were randomized to treatment with sertraline or interpersonal psychotherapy adapted to PTSD (IPT-PTSD) and were followed for 14 weeks. Sertraline dosage ranged from 50 to 200 mg/daily, depending on clinical presentation and tolerance. IPT-PTSD was delivered in 14-weekly 50-min sessions. Participants were clinically evaluated at baseline, week 2, week 4, week 8, and week 14. After 14 weeks, participants received treatment as usual: antidepressants, antipsychotics, hypnotics, and/or psychotherapy until completing one year of follow-up (Fig. 1).

### Measures

The Clinician-Administered PTSD Scale for DSM-5 (CAPS-5) is a structured diagnostic interview containing a 30-item questionnaire capable of assessing the frequency and intensity of PTSD symptoms and the variables associated with the trauma using a frequency/severity scale varying from 0 to 4. A large-scale psychometric study showed strong evidence of its validity and reliability as a measure for the symptoms of PTSD. A validated Brazilian Portuguese language version was used [23, 24].

The Beck Depression Inventory–Second Edition (BDI-II) is a self-reported instrument composed of a 21-item questionnaire measuring clinical depression. The participant must evaluate each question regarding depression symptoms on a 0–4 severity scale. The score consists of the sum of the individual items classifying the severity of the depression as minimum 0–13, mild 14–19, moderate 20–28, or severe 29–63. A validated Portuguese language version was used [25, 26].

The Pittsburgh Sleep Quality Index (PSQI) is a 19-item self-reported measure that assesses seven components of sleep quality, including sleep latency, duration, efficiency, disturbances, use of sleep medication, and daytime dysfunction. Each component is rated on a 0–3 scale referring to the frequency of each disturbance, and the sum of the components provides a global score ranging from 0 to 21. A PSQI global score higher than 5 indicates clinical sleep disturbance. A validated Portuguese language version was used [27, 28].

The Insomnia Severity Index (ISI) is a 7-item self-report questionnaire measuring the patient’s self-perception of insomnia. The ISI targets the subjective symptoms and consequences of insomnia and assesses the severity of sleep onset, sleep maintenance, early morning awakening problems, sleep dissatisfaction, interference of sleep difficulties with daytime functioning, noticeability of sleep problems by others, and distress caused by sleep difficulties. The total score ranges from 0 to 28; 0–7 indicates the absence of insomnia, 8–14 is subthreshold, 15–21 is moderate, and 22–28 is severe [29, 30].

### Polysomnography

Polysomnography is the gold standard for objectively measuring sleep. Participants underwent one night of polysomnography recording at baseline and one night at the one-year follow-up. PSG recordings were conducted in the Instituto do Sono-AFIP at the Universidade Federal de Sao Paulo. Recordings were conducted using an Embla (Embla N7000, Embla Systems, Inc., Broomfield, CO, USA), and all recordings included a six-channel electroencephalogram (EEG) (F4-M1, C4-M1,02-M1, F3-M2, C3-M2, and 01-M2), a two-channel electrooculogram, a four-channel electromyograph (EMG) (electrodes submental and tibial) and a one-channel electrocardiograph (D2-modified). Airflow was detected using a thermocouple and nasal cannula. Respiratory effort was assessed using inductance plethysmography belts. Snoring, body position, blood oxygen saturation, and pulse were evaluated using Embla® sensors (Embla Systems Inc.). All PSGs were performed and scored by two technicians following sleep studies guidelines and were reviewed by a sleep medicine physician. Sleep stages, EEG arousals, and leg movements were scored according to established criteria [31]. Apnea was defined as complete or close to complete airflow cessation for ≥ 10 s, and hypopnea was identified as an evident reduction in the breathing amplitude (at least 30% below the baseline) for ≥ 10 s accompanied by either an EEG arousal or a blood oxygen saturation drop of ≥ 3% [32, 33].

The following sleep variables were determined: REM sleep latency, sleep onset latency; total sleep time, wake after sleep onset, sleep efficiency, and the percentages of total sleep composed of N1, N2, N3, REM sleep, arousal index (number of arousals per hour), periodic limb movements index with and without arousal, number of limb movements per hour with and without arousal, apnea-hypopnea index, number of apneas, and hypopneas per hour. In our study, we included the following variables in the analysis: total sleep time (TST), arousal index (AI), and slow-wave sleep (N3%) since research on sleep and inflammation have associated sleep duration, sleep fragmentation, and slow wave sleep with inflammation [8–12]. We categorized each of the sleep variables into TST (< and > 6 h), N3% (< and > 20), and AI (< and > 10) to assess the effect of sleep architecture on inflammatory markers.

### Blood collection and cytokine assays

Blood samples were collected at approximately 7 AM on the day following the PSG recordings. The inflammatory markers were assayed from overnight fasting serum samples. The serum was stored at − 80 ºC. The Milliplex Map® panel based on the Luminex Map® technology was used to measure interleukin (IL)-1β, IL-6, and tumor necrosis factor (TNF)-α, and highly sensitive enzyme-linked immunosorbent was used to measure the C-reactive protein (CRP) levels. All procedures were performed following the manufacturers’ guidelines. After completing one year in the study, participants repeated blood collection and analysis procedures.

The minimum detectable concentrations for IL-1β, IL-6, TNF-α, and CRP were 0.8 pg/ml, 0.9 pg/ml, 0.7 pg/ml, and 0.9 ng/ml, respectively. Samples were sent for analysis at baseline (T1) and one-year follow-up (T2). All analyses were performed in duplicate. Samples were kept for a maximum of two and a half years for the first analysis and three years for the second analysis. All procedures were performed following the manufacturers’ guidelines.

### Statistical analysis

Statistical analyses were performed using SPSS software version 24.0, with an alpha level of 0.05 for all analyses. The Kolmogorov-Smirnov test was used to analyze continuous data distributions. Log transformation was applied if the data did not fit the normality assumption. A generalized linear model was used to analyze clinical characteristics between groups (PTSD group at baseline and after one-year follow-up). Variables were expressed as means and standard deviations. Fisher exact test or Chi-Square test was used to compare frequencies. Multiple linear regression analysis was used to estimate the association of inflammatory markers’ delta values (T2-T1) with clinical and sleep assessments’ delta values (T2-T1). The final model was obtained from preliminary analysis using bivariate Pearson correlations. The best-fit model was chosen using the Wald method. The Durbin-Watson test was used for autocorrelations, and the variance inflation factor (VIF) and tolerance were performed to determine the presence of multicollinearity. We calculated tolerance and VIF values to evaluate multicollinearity between variables, with tolerance > 0.2 and VIF < 10 indicating no collinearity among the independent variables. Regarding the extreme (outliers) values of the concentrations of the cytokines, we used Z-score to determine outliers. The cutoff values for finding outliers were a Z-score of +/- 3 or more. Participants with outliers’ values were excluded from the analysis. The generalized estimating equation (GEE) was used for data analysis at baseline (T1) and after a one-year (T2) follow-up.

## Results

Clinical and sleep assessments, PSG parameters, and inflammatory markers levels collected at baseline and at one-year follow-up are displayed in Table 1. The mean age of the sample was 24.5 ± 6.8 years, and the mean body mass index was 24.5 ± 4.8 kg/m^2^. The mean time between the sexual assault and baseline evaluation was 2.4 ± 1.7 months.

The patients showed a significant increase in the levels of inflammatory markers IL-1β (p < 0.001), TNF-α (p < 0.001), and CRP (p < 0.001), despite improvement in PTSD (p < 0.001) and depression (p < 0.001) symptoms from baseline through one year follow-up. Regarding sleep assessments, there was an improvement in sleep quality (PSQI, p = 0.001) and insomnia severity (p < 0.001).

**Table 1:** Clinical sleep assessments, PSG parameters, and inflammatory markers of the PTSD group at baseline (T1) and one-year follow-up (T2)

	T1 (N = 57) mean ± SD	T2 (N = 29) mean ± SD	p
**Clinical assessments**			
BDI	27.68 ± 12.55	11.65 ± 11.61	**< 0.001**
CAPS-5	42.44 ± 9.31	20.27 ± 18.30	**< 0.001**
**Sleep assessments**			
PSQI	10 ± 3.55	6.81 ± 5.22	**0.001**
ISI	15.07 ± 6.08	7.73 ± 7.92	**< 0.001**
**PSG parameters**			
TST	340.11 ± 62.51	336.30 ± 111.01	0.80
N3%	24.12 ± 7.14	21.88 ± 9.86	0.21
AI	10.61 ± 4.15	13.44 ± 8.48	0.06
**Inflammatory markers**			
IL-1β	1.38 ± 1.10	1.88 ± 0.62	**< 0.001**
IL-6	1.75 ± 1.64	2.15 ± 1.56	0.32
TNF-α	6.81 ± 2.55	11.54 ± 5.20	**< 0.001**
CRP	0.29 ± 0.54	2.75 ± 1.62	**< 0.001**

GEE model, p < 0.05

BDI = Beck Depression Inventory; CAPS-5 = Clinician-Administered PTSD Scale; PSQI = Pittsburgh Sleep Quality Index; ISI = Insomnia Severity Index; TST = Total Sleep Time; N3% = N3 sleep percentage; AI = Arousal Index; IL1β = Interleukin1β; IL-6 = Interleukin 6; TNF-α = Tumor necrosis factor α; CRP = C-reactive protein; T1 = Baseline; T2 = One year

Regression analysis revealed a significant association between arousal index (AI) and IL-6 score change from T2 - T1. Patients with greater arousal index values were associated with higher IL-6 values (Table 2). This finding suggests that sleep fragmentation is associated with IL-6 enhancement.

Regarding CRP (Table 2), there was a significant association between insomnia severity (ISI) and CRP score change from T2-T1, indicating that patients with worse insomnia showed higher CRP levels. We found no association between clinical and sleep measurements and IL-1β and TNF-α levels (Table 3). None of the inflammatory markers were associated with PTSD symptoms (CAPS-5), depressive symptoms (BDI), and sleep quality (PSQI).

**Table 2:** Multiple regression analysis with inflammatory markers (IL-6 and CRP) as dependent variables

	delta IL-6 as dependent variable					delta CRP as dependent variable				
Independent variables	Coefficient **B**	t	p	95% confidence interval		Coefficient B	t	p	95% confidence interval	
				Lower bound	Upper bound				Lower bound	Upper bound
**Constant**	-1.613	-0.754	0.462	-6.173	2.946	0.272	0.153	0.880	-3.489	4.032
**Delta TST**	0.000	-0.052	0.959	-0.006	0.006	0.001	0.588	0.565	-0.004	0.006
**Delta N3%**	0.052	1.119	0.281	-0.047	0.150	0.007	0.190	0.851	-0.069	0.083
**Delta AI**	0.250	2.503	**0.024**	0.037	0.463	0.132	1.592	0.131	-0.044	0.307
**BMI**	0.067	0.850	0.408	-0.101	0.236	0.095	1.445	0.168	-0.045	0.235
**Delta ISI**	0.071	1.159	0.265	-0.060	0.202	0.146	2.824	**0.012**	0.036	0.255
**Delta PSQI**	-0.218	-1.594	0.132	-0.510	0.073	-0.021	-0.186	0.854	-0.264	0.221
**Delta CAPS**	0.006	0.204	0.841	-0.054	0.065	-0.011	-0.493	0.629	-0.061	0.038
**Delta BDI**	0.007	0.249	0.807	-0.052	0.066	-0.035	-1.501	0.153	-0.084	0.014
R^2^ = 0.31						R^2^ = 0.52				

Abbreviations: TST = Total Sleep Time; N3% = N3 sleep percentage; AI = Arousal Index, BMI = Body mass index, BDI = Beck Depression

Inventory, PSQI = Pittsburgh Sleep Quality Index; ISI = Insomnia Severity Index; CAPS-5 = Clinician-Administered PTSD Scale

CRP = C reactive protein; IL-6 = interleukin 6, Delta = delta values (score change from T2-T1)

**Table 3:** Multiple regression analysis with inflammatory markers (**IL-1β, TNF-α**) as dependent variables

	delta IL-1β as dependent variable					delta TNF-α as dependent variable				
Independent variables	Coefficient B	t	p	95% confidence interval		Coefficient B	t	p	95% confidence interval	
				Lower bound	Upper bound				Lower bound	Upper bound
**Constant**	0.377	0.555	0.587	-1.070	1.823	-0.848	-0.125	0.902	-15.204	13.508
**Delta TST**	-0.002	-1.805	0.091	-0.004	0.000	0.005	0.501	0.623	-0.015	0.024
**Delta N3%**	0.009	0.653	0.524	-0.021	0.040	0.026	0.186	0.855	-0.266	0.317
**Delta AI**	0.060	1.736	0.103	-0.014	0.133	0.367	1.163	0.262	-0.302	1.036
**BMI**	0.007	0.280	0.783	-0.047	0.061	0.218	0.867	0.399	-0.316	0.753
**Delta ISI**	0.012	0.576	0.573	-0.033	0.057	0.209	1.061	0.305	-0.208	0.626
**Delta PSQI**	-0.031	-0.715	0.485	-0.125	0.062	-0.509	-1.164	0.261	-1.435	0.418
**Delta CAPS**	-0.004	-0.396	0.698	-0.022	0.015	0.094	1.056	0.307	-0.094	0.282
**Delta BDI**	0.007	0.742	0.469	-0.013	0.027	-0.078	-0.884	0.390	-0.266	0.109
R^2^ = 0.33						R^2^ = 0.21				

Abbreviations: TST = Total Sleep Time; N3% = N3 sleep percentage; AI = Arousal Index; BMI = Body Mass Index; BDI = Beck Depression Inventory; PSQI = Pittsburgh Sleep Quality Index; ISI = Insomnia Severity Index; CAPS-5 = Clinician-Administered PTSD Scale; IL-1 = Interleukin-1; TNF-α = Tumor necrosis factor α; Delta = delta values (score change from T2-T1)

### GEE analysis

#### Inflammatory markers and total sleep time (TST): TST < and > six hours (Table 4)

We found a significant interaction between sleep duration and CRP levels. Participants who slept more than 6 h per night showed greater CRP levels when compared to women who slept less than 6 h after controlling for body mass index, depression, and PTSD severity.

#### Inflammatory markers and slow-wave sleep (SWS): N3% < 20 and > 20 (Table 4)

There were significant interaction effects between SWS and IL-1β and TNF-α levels. The N3% < 20 group showed increased levels of IL-1β and TNF-α than the N3% > 20 groups throughout one year of follow-up. Participants with lower SWS had higher IL-1β and TNF-α levels after adjustment for confounders. Regarding CRP levels, we found a trend toward significance in the association with lower SWS (p = 0.06) after adjustment.

#### Inflammatory markers and arousal index (AI): AI < 10 and > 10 (Table 4)

No significant interaction effects existed between sleep fragmentation (expressed in AI) categories and inflammatory levels throughout the one-year study.

**Table 4:** Comparison of dichotomized total sleep time, slow wave sleep % and arousal index, and inflammatory markers at baseline and one-year follow-up

Total Sleep Time		TST < 6 h	TST > 6 h	p	p1
**IL-1β**	T1	1.25 ± 0.54	1.23 ± 0.70	0.30	0.39
	T2	1.91 ± 0.93	1.67 ± 0.62		
**IL-6**	T1	1.60 ± 1.17	1.52 ± 1.48	0.70	0.07
	T2	1.89 ± 2.26	1.97 ± 2.03		
**TNF-α**	T1	6.86 ± 3.67	6.72 ± 3.60	0.14	0.47
	T2	10.40 ± 10.54	12.77 ± 9.68		
**CRP**	T1	0.16 ± 0.23	0.28 ± 0.54	0.91	**< 0.001**
	T2	2.18 ± 2.88	3.34 ± 23.04		
**Slow-Wave Sleep %**		**N3% < 20**	**N3% > 20**	**p**	**p1**
**IL-1β**	T1	1.64 ± 0.70	1.18 ± 0.46	0.24	**0.019**
	T2	1.87 ± 0.85	1.26 ± 0.54		
**IL-6**	T1	1.73 ± 1.64	1.87 ± 2.65	0.30	0.17
	T2	2.03 ± 2.10	2.44 ± 0.78		
**TNF-α**	T1	6.73 ± 2.90	7.23 ± 4.68	0.67	**0.018**
	T2	11.63 ± 5.99	11.54 ± 3.67		
**CRP**	T1	0.28 ± 4.13	0.11 ± 0.07	0.01	0.06
	T2	2.95 ± 2.65	0.23 ± 0.31		
**Arousal Index**		**AI < 10**	**AI > 10**	**p**	**p1**
**IL-1β**	T1	1.27 ± 0.62	1.20 ± 0.62	0.74	0.31
	T2	1.79 ± 0.85	1.81 ± 0.78		
**IL-6**	T1	1.38 ± 0.70	1.78 ± 1.79	0.96	0.08
	T2	2.11 ± 2.03	1.61 ± 1.87		
**TNF-α**	T1	6.79 ± 3.67	6.81 ± 3.67	0.54	0.22
	T2	11.14 ± 9.40	12.30 ± 11.70		
**CRP**	T1	0.21 ± 0.31	0.19 ± 0.46	0.27	0.12
	T2	3.16 ± 2.57	1.94 ± 3.67		

GEE model, p and p1 = < 0.05

Abbreviations: IL-1β = Interleukin 1β; IL-6 = Interleukin 6; TNF-α = Tumor necrosis factor α; CRP = C-reactive protein; TST = Total sleep time; N3% = N3 sleep percentage; AI = Arousal Index; GEE = Generalized estimating equation; p = p-value; p1 = p adjusted for BMI, BDI and CAPS-5; T1 = baseline, T2 = one year follow-up

## Discussion

The current study examined subjective and objective sleep measurements and levels of inflammatory cytokines following one year of PTSD treatment in a sample of young women. Our results showed a significant increase in the pro-inflammatory markers despite PTSD symptoms improvement after one year of follow-up, suggesting that neurobiological processes may advance independently of clinical improvement. Nevertheless, we found a significant association between greater sleep fragmentation and IL-6, decreased SWS and TNF-α, and greater insomnia severity, sleep duration, and CRP.

These results are consistent with some previous studies on inflammation and sleep. Two studies have found an association between sleep and IL-6. Vgontzas and colleagues (2019) reported that IL-6 levels had a negative association with the amount and depth of sleep; sleep deprivation was associated with increased daytime IL-6 levels, while good sleep quality was associated with decreased IL-6 [34]. Hong and colleagues (2005) evaluated the association of IL-6 with sleep architecture in healthy subjects and suggested that daytime IL-6 levels were negatively associated with sleep efficiency and slow-wave sleep [35]. Furthermore, in a study that investigated the effects of two hours of sleep deprivation per night found increased TNF-α in healthy men but not women [36].

Previous investigations of sleep duration and CRP have produced mixed results. Some studies have found an association between increased CRP and short sleep duration [10, 37], while others have found increased CRP and long sleep duration [38, 39]. In our study, levels of CRP were significantly higher in women who slept more than 6 h per night, compared to women who slept less than 6 h. Consistent with our findings, studies showed that each additional hour of sleep duration was associated with an 8% increase in CRP levels [39] and longer sleep duration was associated with higher levels of CRP and IL-6. These associations remained after adjustment for waist circumference, diabetes, heart disease, and depressive symptoms [40]. A large-scale study found higher CRP levels in women who slept 5 h or less but no association between sleep duration and CRP was observed in men [41]. Indeed, some evidence suggests that sex may influence the inflammatory response to sleep impairments [41–43].

The mechanisms that might explain the relationship and directionality between sleep and inflammation are complex and are not fully understood. Physiological sleep is associated with a decline in circulating catecholamines, with lower levels of noradrenaline and adrenaline during sleep [44]. Brief sleep deprivation or fragmentation is usually associated with mild and temporary increased sympathetic activity. However, chronic sleep deprivation can promote the activation of the stress systems, and a progressive and gradual increase in catecholamine levels [45]. These neurobiological changes may not be immediately evident; however, studies have shown that chronic sleep deprivation can gradually affect the reactivity of the neuroendocrine system, increasing vulnerability or maintenance of stress-related disorders [46]. Therefore, chronic sleep impairments in PTSD can cause gradual changes in the central nervous system and affect neuroendocrine system reactivity, and pro-inflammatory response over time. These neurobiological processes may advance independently of PTSD symptoms. In the reverse direction, increased levels of inflammatory mediators may act directly on neurons in the brain to further interrupt sleep [47].

The present study has limitations. Because our participants were sexually assaulted women and had severe PTSD symptoms, the high dropout rate observed in the follow-up may be due to feelings of shame, fear of stigma, or avoidance of exposure to painful memories, which is one of the symptoms of PTSD. An additional limitation, the objective sleep measures were depicted from one night of PSG assessments, which might not represent the participants’ sleep duration. However, an epidemiological study examining risk factors for chronic insomnia based on one night of PSG showed that their results were consistent with other epidemiological studies regarding objective sleep duration [48]. Another limitation is that neither socioeconomic status nor randomized treatment (sertraline vs. psychotherapy) was adjusted for possible confounders. Lastly, we examined inflammatory markers based on a single blood sample drawn at enrollment and after one year. Multiple and sequential blood testing could better demonstrate the trend toward the increase in inflammatory markers.

Our strengths include a longitudinal study design in young women with PTSD following sexual assault, subjective sleep measurements, in-laboratory PSG monitoring, and measures of several pro-inflammatory markers.

## Conclusion

In summary, our results suggest that chronic sleep restriction and fragmentation may be associated with a gradual and persistent alteration in the immune mediators, resulting in a progressive inflammatory process, which, in turn, may be a risk factor for several pathologies such as cardiovascular and neurodegenerative diseases in PTSD women.

Prospective studies investigating the association between the immune, neuroendocrine, and central nervous systems in PTSD are essential for better understanding of sleep disorders’ pathophysiology. Our findings suggest that improving the assessment and treatment of sleep disturbances in PTSD women may reduce morbidity risk and PTSD symptoms severity.

## Data Availability

The datasets used or analyzed during the current study are available from the corresponding author upon reasonable request.

## List of abbreviations

ACTH: Adrenocorticotropic hormone
AI: Arousal index
BAI: Beck Anxiety Inventory
BDI: Beck Depression Inventory
BMI: Body mass index
CAPS-5: Clinician-Administered PTSD Scale
CRH: Corticotropin-releasing hormone
CRP: C-reactive protein
DSM-5: Diagnostic and statistical manual of mental disorders
EEG: Electroencephalogram
ELISA: Enzyme-Linked-Immuno-Sorbent Assay
EMG: Electromyograph
GEE: Generalized estimating equation
GLM: Generalized linear model (GLM)
HPA: Hypothalamic-pituitary-adrenal
IL-1β: Interleukin-1β
IL-6: Interleukin-6
IPT-PTSD: Interpersonal psychotherapy adapted to PTSD
ISI: Insomnia Severity Index
M: Mean
MDD: Major Depressive Disorder
MINI: Mini-International Neuropsychiatric Interview
N3%: Percentage of total sleep composed of N3
NF-kB: Nuclear factor kappa B
PSG: Polysomnography
PSQI: Pittsburgh Sleep Quality Index
PTSD: Post-traumatic stress disorder
SD: Standard deviation
SE: Standard Error
SWS: Slow-wave sleep
TST: Total sleep time
TNF-α: Tumor necrosis factor α
VIF: Variance inflation factor
